# FPIES: Data for Germany in international comparison 

**DOI:** 10.5414/ALX02363E

**Published:** 2022-10-05

**Authors:** Sunhild Gernert, Antje Finger, Lars Lange

**Affiliations:** 1 GFO Kliniken Bonn, St. Marien-Hospital, Department of Pediatrics, Bonn, and; 2 Helios University Hospital Wuppertal, Center for Pediatric and Adolescent Medicine, Wuppertal, Germany

**Keywords:** FPIES, non-IgE-mediated food allergy, triggering foods, complementary feeding, tolerance, Germany

## Abstract

Food protein-induced enterocolitis syndrome (FPIES) is a rare, non-IgE-mediated food allergy. The triggering foods differ significantly from the typical triggers of an IgE-mediated food allergy. Until recently, there were no data on triggers of FPIES in Germany. In order to create an advisory basis for the care of German patients, a large multicenter study was initiated and published at the end of 2021. This revealed clear differences in international comparisons. The most frequent triggers for FPIES in Germany are cow’s milk, fish, vegetables, and meat. Most children (84%) react to only one food. The prognosis is usually good, depending on the trigger. Regional data should be used for counseling patients with FPIES. Specific recommendations for this are given in this article.

## Background 

Food protein-induced enterocolitis syndrome (FPIES) is a rare, non-IgE-mediated food allergy that has been described since the 1980s and is increasingly better understood. It most commonly presents in infancy and early childhood. In the majority of cases, patients present with repetitive vomiting, pallor, diarrhea, and lethargy up to hypovolemic shock occurring 1 – 4 hours after ingestion of the triggering food. According to the diagnostic criteria of 2017 [1] (Table 1), two different clinical pictures of FPIES should be distinguished: acute FPIES, in which symptoms always reproducibly occur only after consumption of the trigger food and chronic FPIES, which usually affects very young infants before the start of complementary feeding. In this case, non-specific symptoms such as failure to thrive, intermittent vomiting, sometimes (bloody) diarrhea, and even sepsis-like pictures with severe dehydration occur. The triggers of chronic FPIES are proteins of formula – usually based on cow’s milk or soy – or allergens transferred via breast milk. 

Triggers of acute FPIES can be any food in the child’s diet (Table 2). Epidemiological data on FPIES in children are limited, depend on the type of FPIES investigated (chronic or acute) and vary regionally. A review of worldwide data identified incidences of 0.015 – 0.7% in children [2]. The authors also summarized the main triggers of FPIES: worldwide, cow’s milk is reported by far the most. The other triggers differ depending on the region in which the study was conducted [3, 4, 5, 6, 7, 8, 9, 10]. 

Since no data on FPIES triggers had been available from Germany so far, the aim of a German retrospective multicenter study in 142 German children was the identification of the most frequent triggers in order to provide relevant feeding advice to families [11]. Furthermore, the development of tolerance depending on the triggering food, the number of triggers, the clinical presentation, and the presence of IgE sensitization to the trigger was investigated. In this study, 130 acute FPIES reactions and 60 chronic ones could be evaluated. 

## Triggers of acute FPIES 

Typical triggers of FPIES differ significantly from IgE-mediated food allergies (Table 2). Physicians and nutritionists need to know for the consultation of patients which triggers exist, whether children tend to react against multiple triggers, and what tolerance development is to be expected. 

Until a few years ago, they could only refer to American and Australian data [3, 4, 5, 6]. These reported soy, rice, oat (USA) or rice, egg, and oat (Australia) [6] as the most frequent triggers of acute FPIES after cow’s milk. With an increasing number of clinical studies also from Europe, the causative foods of acute FPIES were found to vary regionally. In Italy, Spain, Greece, the United Kingdom, and Sweden, fish was the most common trigger from the solid food group [7, 8, 9, 12, 16]. In France, egg [10], and in another study from the British Isles, fruit and vegetables [13] were mentioned as the most frequent triggers. Other common triggers in the European studies were oats, rice, and egg. 

**Figure 1:**
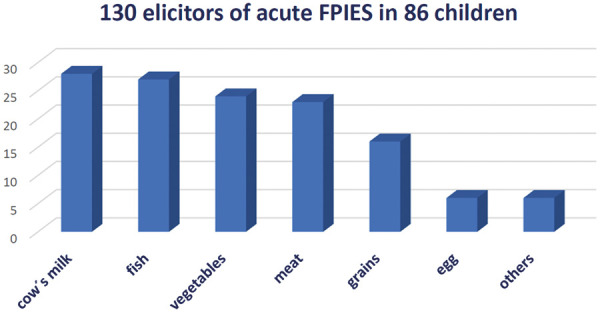
Elicitors of acute FPIES in German infants and children (modified based on [11]).

**Figure 2:**
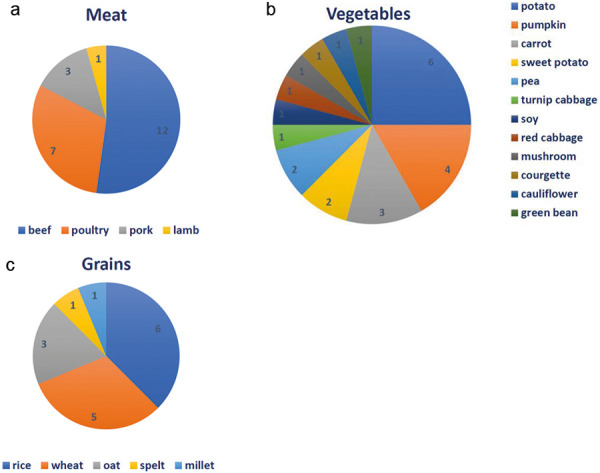
Frequencies of different food triggers in acute FPIES in Germany in the food groups meat (a), vegetables (b), and grains (c) [11].

In Germany, the pattern of triggers was different (Figure 1, Figure 2): most common triggers of acute FPIES were also cow’s milk and fish, followed by vegetables, meat, and grains (Figure 1). Salmon was the most commonly cited trigger for fish FPIES. Reactions to meat occurred most frequently after ingestion of beef, followed by poultry and pork. Potato, pumpkin, and carrot were the most common triggers of an FPIES reaction to vegetables. Rice and wheat were equally frequent triggers in reactions to grains (Figure 2). 

## Relevance of early complementary feeding 

It is noticeable, that the triggering foods are often among those introduced early into infants’ diet. Many infants in Germany receive potato as one of the first solid foods in a vegetable-potato mash. Beef and salmon are also introduced early according to current feeding recommendations; they were the most common solid foods triggering FPIES in our cohort. Soy, which is one of the most common triggers of FPIES outside Europe, is rarely part of early complementary feeding in Germany and was observed as a trigger in only 1 case in our study. There is evidence that the timing of introduction of a food determines whether FPIES reactions subsequently occur. In the United States, Lopes et al. [14] noted a remarkable increase of cases with FPIES to peanut since the early introduction of peanut was implemented into the diet of infants to prevent IgE-mediated allergy. Previously, the EAT study [15] showed that the very early introduction of egg resulted in several cases of FPIES to egg. The regionally varying timing of the introduction of potential allergens into the diet of infants is part of the explanation for the global differences in FPIES triggers among other allergen- and patient-specific factors. 

## Nutritional recommendations depending on dietary habits 

In the international consensus guidelines, recommendations for the introduction of complementary feeding in FPIES are given on the basis of the data available primarily from the USA and Australia [1]. There, foods with a high risk for the development of acute FPIES are listed, which should initially be avoided in the complementary feeding when FPIES has been diagnosed. For example, foods with moderate or high risk for developing FPIES are pea, oat, hen’s egg, apple, pear, and soy. These foods are hardly or not at all relevant as triggers in Germany. Triggers that are common in Germany, such as beef, potato or pumpkin, are on the other hand classified internationally as those with moderate or low risk. 

Assuming that the trigger profile depends on local dietary habits and recommendations for complementary feeding, it seems questionable to classify different foods into different risk categories. There is a risk that the alternatively chosen foods may also trigger a reaction if they are introduced during the time window in which a child’s organism with a predisposition to develop FPIES is susceptible. 

## Number of triggering foods 

For the counseling of patients’ parents, it is important to know that the majority of children react to only 1 food. In our study, this was true for 84% of all patients and, if only the children who reacted to cow’s milk were analyzed, even 90%. This also corresponds to the observations of other European studies where the proportion of children with only 1 FPIES trigger ranged from 70 to 94% [7, 8, 9, 10, 12]. In the German study, only 16% of patients had multiple triggers, of which 5% had more than 3 different triggers. Children who had their first reaction to a solid food reacted to additional triggers in 25% of the cases. 

Those with multiple triggers showed only partial reactions to foods of the same food group. There were 4 children who reacted to more than 1 type of meat and 6 children who reacted to both meat and fish. The trigger combination of beef and milk was observed in 5 children. Two children reacted to more than 1 allergen of a vegetable family (1 child to cucurbits, 1 child to cabbage). Therefore, the question of cross-reactivity arises mainly in cases of close biological relationship of the allergens, such as different types of fish, meat, or milk and beef. In other children with multiple triggers, there was no apparent relationship between these. Thus, further reactions are rare, but also unpredictable. 

This point should be discussed in detail during the consultation. For the majority of patients, complementary feeding can be introduced without restriction (Table 3). 

## Tolerance development 

Data on tolerance development in patients with FPIES are comparable worldwide [8, 9, 10, 11, 16]: those who reacted to cow’s milk became tolerant earliest, while those who reacted to solid foods did so later. In the German cohort, a difference in tolerance development was also found between children initially presenting with chronic versus acute FPIES: children with chronic FPIES were tolerant after a mean of 16.5 months, whereas those with acute FPIES were tolerant after 19.5 months [11]. 

For cow’s milk FPIES, the mean age of tolerance in the above-mentioned studies was between 16 and 24 months. There is only one study by Caubet et al. [5] that found a significant later age of tolerance to milk in U.S. children (5 and 13 years) depending on whether the children had developed specific IgE against milk or not. The significant differences to the very homogeneous European data may be explained by the cautious indication for food challenges in the USA. 

Of the patients with solid foods as triggers, the children in our study with reactions to grains developed tolerance earlier compared to children who reacted to fish and meat. The age of tolerance of children with reactions to vegetables was in between the two groups. Data on tolerance development to hen’s egg vary: in France, children were tolerant after a mean of 2.3 years [10], and in Germany and Italy [11, 16] after ~ 5 years. Children who developed reactions to multiple triggers did not show delayed tolerance development [11]. 

Thus it should be noted for the consultation that the timing of tolerance depends primarily on the trigger, but not on the number of triggers or an existing sensitization and is expected to be earlier for milk than for solid foods. Food challenges in order to prove tolerance should be performed for cow’s milk, cereal, and vegetable FPIES after ~ 1 – 2 years, for meat and fish every 3 – 5 years. 

## Chronic FPIES 

Although infants with chronic FPIES often initially present in a poor condition, the prognosis is very good. The trigger of chronic FPIES in Germany was almost exclusively cow’s milk or cow’s milk formula [11]. Four children developed chronic FPIES under exclusive breast milk feeding. In 1 of the infants with FPIES triggered by breast milk, food challenge later confirmed cow’s milk proteins transferred via breast milk as the trigger. Ullberg et al. [9] showed in a larger number of infants that symptoms of chronic FPIES were triggered by various food proteins transferred via breast milk. In this study, mainly cow’s milk, but rarely also rice, oat, and soy were identified as triggers in breast milk. 

In our cohort, only 7% out of the 60 children with chronic FPIES showed reactions to additional triggers further on. Additionally, they were tolerant earlier than the children with acute FPIES. 

Apart from avoiding cow’s milk protein and providing a replacement diet based on an extensive hydrolysate or amino acid formula, no further nutritional restrictions are required. A first food challenge to prove tolerance can be conducted between the end of the first and the end of the second year of life. 

## Atypical FPIES 

FPIES belongs to the non-IgE-mediated food allergies. Nevertheless, some patients develop specific IgE against their trigger. This condition is called atypical FPIES. Not many studies systematically investigate sensitization in patients with FPIES. A sensitization rate of 16% was reported in Spain [8], and as high as 24% in the United States [5], whereas in Sweden it was only 4% [9]. In our study, 14% of the children were sensitized. Similar to the other reports, the allergen to which sensitization was present was mostly cow’s milk (75% of the German children). We observed that specific IgE to the trigger was present in some cases throughout the course of the disease and disappeared or developed during the course in other children. Knowledge of sensitization is important because the reaction pattern may change. Sopo et al. [17] described 2 children who changed from an FPIES-typical reaction to typical IgE-mediated symptoms during the course of the disease. This was also observed twice in the German cohort. It is therefore reasonable to perform a sensitization test initially, but especially before planning a food challenge. In contrast to the data of Caubet et al. [5], we could not confirm a negative influence of IgE sensitization on tolerance development. 

## (Re-)introduction of food 

When introducing a new food in children with known FPIES, it is important to know that reactions do not always occur at the first contact. We described 3 children who tolerated their FPIES trigger in the food challenge and reacted to the same trigger some days later at home [17]. In an Australian study, it was recorded that only 61% of patients reacted to the first exposure to the food, some only to the fourth consumption [18]. To date, there is no explanation for this observation. It is possible that regular and prompt consumption of the food after a negative challenge starting the next day is crucial. The 3 children we described paused the consumption for several days after the negative food challenge. 

## Management of reactions 

Once FPIES is diagnosed and the trigger is known, reactions rarely occur. Because usually several grams of the allergen are required to trigger a reaction [17], “trace avoidance” is not recommended. Reactions presenting as acute FPIES must be treated primarily with fluid substitution, orally or intravenously with balanced electrolyte solution (10 – 20 mL/kg), depending on symptom severity. There is no evidence for the effectiveness of treatment with steroids (e.g., prednisolone 1 – 2 mg/kg), but administration is recommended [1]. There is a benefit for parenteral administration of ondansetron at 0.15 mg/kg, which is approved from 6 months of age, but not for the treatment of FPIES reactions. Therefore, both information about off-label use and ECG recording to rule out long-QT syndrome are recommended before use. FPIES emergency cards are available from the German Allergy and Asthma Association (DAAB e.V.). In these, the clinical picture FPIES is briefly described for first-care physicians, the individual triggers are listed, and emergency medication is suggested. 

## Summary 

Since the end of 2021, data on FPIES in German children have been available for the first time. With regard to the triggers cow’s milk, fish, and vegetables, similarities can be seen with other European countries. On the other hand, there are differences in foods such as egg or oat, which are less important as triggers in Germany. Among the most common triggers were foods introduced early in the infant’s diet. It is possible that the time of exposure determines whether a food becomes a potential FPIES trigger or not. Comparable to data from other European studies, the majority of affected children from Germany had only 1 FPIES trigger. Because the trigger profile depends in parts on local dietary habits, we do not consider nutritional restrictions based on the risk table of the international consensus guideline 2017 to be justified. 

Like it had turned out in other studies, tolerance was achieved at different ages depending on the trigger in the German study: first for cow’s milk, followed by grains and later vegetables. For meat and fish, tolerance occurred significantly later. Multiple triggers were not associated with delayed tolerance development. In contrast to previous publications, we did not find delayed development of tolerance in children with IgE sensitization to the triggering food. Food challenges in order to prove tolerance can be performed in milk FPIES between the first and second birthday and should be planned individually depending on the trigger. However, larger studies are necessary to generate more reliable data. 

## Funding 

None. 

## Conflict of interest 

Sunhild Gernert, Antje Finger, and Lars Lange declare no conflicts of interest. 

**Table 1.** Diagnostic criteria for patients presenting with possible FPIES (according to Nowak-Wegrzyn et al. [1]).

Acute FPIES	
**Major criterion:** Vomiting in the 1- to 4-hour period after ingestion of the suspect food and the absence of classic IgE-mediated allergic skin or respiratory symptoms	**Minor criteria (3 or more):** 1. A second (or more) episode of repetitive vomiting after eating the same suspect food 2. Episode of repetitive vomiting 1 – 4 hours after eating a different food 3. Extreme lethargy with any suspected reaction 4. Marked pallor with any suspected reaction 5. Need for emergency room visit with any suspected reaction 6. Need for intravenous fluid support with any suspected reaction 7. Diarrhea within 24 hours (usually 5 – 10 hours) 8. Hypotension 9. Hypothermia
Chronic FPIES	
**Severe presentation:** When the offending food is ingested on a regular basis (e.g., infant formula), intermittent but progressive vomiting and diarrhea (occasionally with blood) develop, sometimes with dehydration and metabolic acidosis. **Milder presentation:** Lower doses of the problematic food (e.g., solid foods or food allergens in breast milk) lead to intermittent vomiting and/or diarrhea, usually with poor weight gain but without dehydration or metabolic acidosis.	The most important criterion for chronic FPIES diagnosis is resolution of the symptoms within days after elimination of the offending food(s) and acute recurrence of symptoms when the food is reintroduced, onset of vomiting within 1 – 4 hours, diarrhea within 24 hours (usually 5 – 10 hours). Without confirmatory challenge, the diagnosis of chronic FPIES remains presumptive.

**Table 2.** Observed triggers of acute FPIES.

Liquid food
Cow’s milk, soy, breast milk
Solid food
**Meat:** beef*, pork*, lamb*, chicken*, turkey **Seafood:** salmon*, cod*, pollack*, tuna*, shellfish, mollusks **Grains:** rice*, wheat*, oat*, spelt*, millet*, corn **Vegetables: **potato*, carrot*, pumpkin*, sweet potato*, pea*, turnip cabbage*, cucumber*, red cabbage*, zucchini*, cauliflower*, champions*, garden bean*, lentil, tomato **Fruit:** banana*, kiwi, apple, pear, avocado, peach, plum, watermelon, strawberry, mango, pineapple, blueberry, apricot, raspberry **Nuts, seeds:** hazelnut*, coconut*, almond*, sunflower seed*, peanut, cashew Hen’s egg*

*Observed in Germany.

**Table 3.** Relevant counseling content for patients with FPIES.

The aim of a counseling session for affected families is to impart knowledge about: – The symptoms – The avoidance of the trigger – The risks of further reactions to reduce both anxiety and the development of eating disorders – The implementation of gradual introduction of complementary feeding
Acute FPIES usually occurs in the first 2 years of life. However, new reactions are also observed in older children and even in adults.
The majority of patients react to only 1 food. Especially in patients with chronic FPIES and FPIES to cow’s milk, further triggers are usually not to be expected. Consequently, complementary food can be introduced without special caution. Symptoms of an acute FPIES reaction should be known, especially in children with chronic FPIES.
In patients with acute FPIES to a solid food, other foods are possible triggers. There is no data on the frequency of different triggers in Germany. In general, almost any food seems to be able to trigger FPIES reactions if it is introduced in a certain time window in susceptible children. Patients who have reacted to meat or fish have an increased risk of reacting to other types of meat or fish.
If a new food has been tolerated, it should be given regularly in the beginning to strengthen tolerance.
In chronic FPIES and FPIES to cow’s milk, tolerance is to be expected already after about 1 year. Food challenges should be performed early for these children.
In patients with reactions to solid food, especially to fish and meat, tolerance development is significantly delayed. Therefore, food challenges are useful after 3 – 5 years.
After proof of tolerance by means of food challenge, the consumption of the former FPIES trigger is first recommended daily and then several times a week in order to prevent a relapse.
Trace avoidance is usually not required.
Processing of the triggering food (cooking, baking, etc.) has no effect on better tolerance
